# Positive Effects of an Anti-Aggression and De-Escalation Training on Ward Atmosphere and Subjective Safety May Depend on Previous Training Experience

**DOI:** 10.3389/fpsyt.2018.00134

**Published:** 2018-04-12

**Authors:** Daniela Fröhlich, Franziska Rabenschlag, Susanne Schoppmann, Stefan Borgwardt, Undine E. Lang, Christian G. Huber

**Affiliations:** Universitäre Psychiatrische Kliniken Basel, Universität Basel, Basel, Switzerland

**Keywords:** ward atmosphere, subjective safety, occupational health, aggression, de-escalation

## Abstract

Anti-aggression and de-escalation (ADE) trainings of health-care professionals working on psychiatric inpatient wards have been shown to increase staff knowledge and confidence, which could be connected with higher subjective safety. Additionally, a potential reduction of aggressive incidents could improve ward atmosphere. Thus, the current study aimed to investigate the effects of ADE training on ward atmosphere and subjective safety. In 2015, an ADE training was established at the Psychiatric University Clinics (UPK), University of Basel. Nursing staff from 22 wards received theoretical and practical training over the course of 5 days. Ward atmosphere and subjective safety were assessed using the Essen Climate Evaluation Schema (EssenCES). A total of 46 people had been assessed in 2012 before training implementation (baseline), and 45 persons in 2016 after implementation. In the 2016 group, 23 people had previously participated in an ADE training, and 22 were first-time participants. Patients’ coherence (*p* = 0.004), subjective safety (*p* = 0.004), and ward atmosphere (*p* = 0.001) were rated significantly higher by first-time ADE training participants compared to baseline, and patients’ coherence (*p* = 0.029) and ward atmosphere (*p* = 0.011) were rated significantly higher by first-time ADE training participants than by nurses with prior ADE training. There were no significant differences regarding any EssenCES ratings by nurses with prior ADE training compared to baseline. ADE training was exclusively connected with higher ratings on most EssenCES scales for first-time participants. This indicates that the positive effects of ADE training may depend on previous training experience.

## Introduction

Aggression is a frequent and clinically relevant problem in psychiatry, endangering the patients, professionals, and the public, and interfering with successful psychiatric therapy (1, 2). Nursing staff is particularly vulnerable to patient violence, with detrimental effects on subjective safety, occupational health, and work satisfaction (3). While ample information exists about the clinical and especially pharmacological management of aggression in psychiatry, current scientific knowledge on the indicated application of involuntary measures, on their effects, and on the prevention of situations requiring involuntary measures is severely lacking (4). Despite weak evidence, current guidelines recommend anti-aggression and de-escalation (ADE) training for health-care professionals to support prevention and adequate management of dangerous situations (5, 6). ADE trainings have been shown to increase staff knowledge, confidence, and de-escalation competence (7). These changes could be connected with the development of a more positive ward atmosphere (8). In addition, there is mixed data concerning the effect of ADE training on the frequency or intensity of aggressive incidents and the occurrence of injuries (7, 9). Improvements in these areas could also be connected with a better ward atmosphere, for example, as ward atmosphere correlates negatively with aggressive incidents (10, 11). Thus, the current study aimed to investigate the effects of an ADE training on ward atmosphere and subjective safety.

## Materials and Methods

Beginning in February 2015, an ADE training adapted from the commercially available RADAR- and ProDeMa-methods (7) was established at the Psychiatric University Clinics (UPK), University of Basel (12). Nursing staff from 22 wards received theoretical and practical training over the course of 5 days in groups of between 12 and 15 participants.

Data collected in 2012 (baseline, group A) and in 2016 (after ADE training) were analyzed in the current study. Baseline data on ward atmosphere on open and closed wards was rated by 46 members of the UPK nursing staff (43.5% female) between June and July 2012 (13). Between February and August 2015, 80 members of the nursing staff working on open and closed wards received ADE training and were followed-up with *via* questionnaire in January 2016; 45 (56.3%) questionnaires were available for analysis. Of these, 23 were completed by people who had previously participated in an ADE training (group B), and 22 by persons who were first-time participants (group C).

General “ward atmosphere,” along with its subdimensions of “patients,” “coherence,” “subjective safety,” and “therapeutic hold,” were examined using the Essen Climate Evaluation Schema (EssenCES), a well-established self-rating scale (10, 13). The EssenCES contains five items per dimension, and each item is rated on a 5-point Likert scale. The subscale “subjective safety” is highly correlated with the frequency of dangerous events (10, 11), and patient ratings are generally similar to staff ratings (8).

Additionally, data on previous ADE training experience and gender were collected. To guarantee the participants’ anonymity, no further information allowing for the potential identification of participants was collected (e.g., information on age or professional experience).

According to current regulations, no approval from an ethics committee was required for this evaluation. Study procedures were carried out in accordance with all local and national regulations and with the Declaration of Helsinki in its latest revision. Data were analyzed using descriptive statistics, χ^2^-tests, *t*-test, and ANOVAs with *post hoc* Scheffé tests, and *p*-values < 0.05 were considered significant.

## Results

Table 1 shows the overall effect of the ADE training.

**Table 1:** Overall effects of anti-aggression and de-escalation (ADE) training.

	2012 (A) *n* = 46	2016 (B + C) *n* = 45	*p*-value
Gender (female)	20 (43.5%)	22 (48.9%)	0.829^1^
**EssenCES subscores**			
Patients’ coherence	9.5 ± 3.5	11.0 ± 2.9	0.032^2^
Subjective safety	7.5 ± 4.9	9.8 ± 4.1	0.011^2^
Therapeutic hold	16.7 ± 1.9	16.0 ± 2.4	0.373^2^
Ward atmosphere	33.0 ± 7.1	36.6 ± 6.8	0.017^2^

*Ward atmosphere, patients’ coherence, subjective safety, and therapeutic hold according to staff assessment in (A) 2012, before implementation of the ADE training, and in 2016, after implementation of the ADE training for (B) staff with prior ADE training experience, and for (C) staff without prior experience. Number (percentage) is shown for nominal variables, and mean ± SD is given for continuous variables. EssenCES: Essen Climate Evaluation Schema*.

*^1^χ^2^-test*.

*^2^t-test*.

There were no significant gender differences between the 2012 and the 2016 groups. Overall, nursing staff reported a significant increase in patients’ coherence, subjective safety, and general ward atmosphere in the time after the ADE training, while there were no significant differences regarding therapeutic hold.

Table 2 shows a subgroup analysis comparing groups A (2012), B (2016 after ADE training, with prior ADE training experience), and C (2016 after ADE training, without prior ADE training experience) to examine the effect of prior ADE training on ward atmosphere ratings.

**Table 2:** Effect of prior anti-aggression and de-escalation (ADE) training.

	2012 (A) *n* = 46	2016 (B) *n* = 23	2016 (C) *n* = 22	*p*-Value	*Post-Hoc* tests
Gender (female)	20 (43.5%)	11 (47.8%)	11 (50.0%)	0.966^1^	
**EssenCES subscores**					
Patients’ coherence	9.5 ± 3.5	9.7 ± 2.9	12.3 ± 2.3	0.003^2^	C > A, C > B
Subjective safety	7.5 ± 4.9	8.4 ± 4.0	11.3 ± 3.7	0.003^2^	C > A
Therapeutic hold	16.7 ± 1.9	15.2 ± 2.6	16.7 ± 2.0	0.046^2^	n.s.
Ward atmosphere	33.0 ± 7.1	33.5 ± 7.0	39.9 ± 5.0	0.001^2^	C > A, C > B

*Ward atmosphere, patients’ coherence, subjective safety, and therapeutic hold according to staff assessment in (A) 2012, before implementation of the ADE training, and in 2016, after implementation of the ADE training for (B) staff with prior ADE training experience and for (C) staff without prior experience. Number (percentage) is shown for nominal variables, and mean ± SD is given for continuous variables. EssenCES, Essen Climate Evaluation Schema. n.s., not significant*.

*^1^χ^2^-test*.

*^2^ANOVA with post hoc Scheffé test*.

Again, there were no significant gender differences. ANOVAs revealed significant between-group differences in patients’ coherence [*F*(2, 84) = 6.2; *p* = 0.003], subjective safety [*F*(2, 88) = 6.1; *p* = 0.003], therapeutic hold [*F*(2, 86) = 3.2; p = 0.046], and ward atmosphere [*F*(2, 84) = 8.0; *p* = 0.001]. In the *post hoc* tests, there were no significant differences regarding any EssenCES subscale between groups A and B. However, patients’ coherence (*p* = 0.004), subjective safety (*p* = 0.004), and ward atmosphere (*p* = 0.001) were rated significantly higher by group C than by group A, and patients’ coherence (*p* = 0.029) as well as ward atmosphere (*p* = 0.011) were rated significantly higher by group C than by group B.

## Discussion

The purpose of this study was to examine the effects of an ADE training on nursing staff ratings of patients’ coherence, ward atmosphere, subjective safety, and therapeutic hold. Our results show that, overall, staff reported higher ratings for patients’ coherence, subjective safety, and ward atmosphere in the time following ADE training. In particular, staff without previous training reported a significantly improved ward atmosphere, subjective safety, and patients’ coherence with respect to baseline ratings. However, staff with previous training experience showed no significant differences in the subgroup analysis. This may explain the conflicting findings regarding the outcome of ADE trainings found in the previous literature. While there is evidence for improved self-confidence as an effect of ADE trainings (7), some studies failed to find effects on nurses’ perceptions toward patient aggression (14). Needham et al. showed that while the severity of aggressive incidents remained unchanged after an ADE training, the subjective severity as assessed by nursing staff declined (15), demonstrating primarily subjective effects of the training. In addition, Blaesi et al. showed that EssenCES scores increased on newly opened wards compared to permanently closed and open wards (13), suggesting subjective effects with respect to change. It is plausible that these effects depend on personal characteristics and previous staff experience.

Our study provides the first evidence for an association between ADE training effects and previous training experience. However, the small sample size, the relatively long time between assessments, and the cross-sectional design limit the interpretability of our findings. In particular, changes in the mix of patient cases, patients’ characteristics, and structural changes in the hospital from 2012 to 2016 may have influenced the present findings. Furthermore, only information on gender was available, and other potential confounding factors, for example, years of professional experience, could not be controlled for. Therefore, the current results should be interpreted with caution, and replication of the findings is needed.

In summary, our study indicates that the positive effects of an ADE training may depend on previous training experience. This information may help to disentangle conflicting results of previous studies, to optimize ADE trainings, and to better address the needs of health-care professionals. Future studies are encouraged and should consider previous ADE training experience as a potential confounder.

## Data Availability Statement

The datasets analyzed in this study can be obtained from the corresponding author on request.

## Ethics Statement

According to current regulations, no approval from an ethics committee was required for this evaluation. Study procedures were carried out in accordance with all local and national regulations and with the Declaration of Helsinki in its latest revision.

## Author Contributions

CH designed the study, and wrote the initial draft of the paper. FR and SS collected the data. DF, FR, SS, and CH analyzed and interpreted the data. CH, DF, FR, SB, SS and UL have contributed to, read, and approved the final version of the manuscript. DF had full access to all the data in the study and takes responsibility for the integrity of the data and the accuracy of the data analysis.

## Conflict of Interest Statement

The authors declare that the research was conducted in the absence of any commercial or financial relationships that could be construed as a potential conflict of interest.
